# Metal urethral stent placement for the management of urethral stricture following phalloplasty: a case report

**DOI:** 10.3389/fruro.2025.1651449

**Published:** 2025-08-11

**Authors:** Hu He, Chuanhua Zhong, Qiang Chen, Yongsheng Lei, Longchao Chen, Lulu Li, Xin Zhang, Jinhong Pan, Heng Zhang

**Affiliations:** Department of Urology, Guiqian International General Hospital, Guiyang, China

**Keywords:** phalloplasty, urethral stricture, stent placement, difficulty urinating, thermo-expandable nickel-titanium shape memory alloy metal stent

## Abstract

**Objective:**

To report the preliminary experience of a case in which a thermo-expandable nickel-titanium shape memory alloy metal stent was utilized for the management of urethral stricture following phalloplasty.

**Methods:**

A 51-year-old male patient underwent lower abdominal island flap phalloplasty due to penile trauma. Postoperative recurrent dysuria occurred, and urethrography revealed stricture at the urethral anastomosis site near the penile root. A thermo-expandable nickel-titanium shape memory alloy metal stent was implanted.

**Results:**

The patient underwent a successful surgical procedure. One day after the surgery, the urethral catheter was removed, and the patient resumed normal urination. After achieving satisfactory outcomes, the patient recovered and was discharged. During regular follow-up visits after discharge, the patient maintained unobstructed urination without weak urine stream or other discomforts.

**Conclusions:**

The placement of a thermo-expandable nickel-titanium shape memory alloy metal stent offers a novel treatment option for patients with urethral stricture following phalloplasty

## 1 Introduction

Urethral stricture following phalloplasty is one of the common complications in patients undergoing phalloplasty due to penile trauma. The stricture primarily occurs at the urethral anastomotic site near the penile root. Due to its unique anatomical characteristics and clinical complexity, the treatment of such condition remains relatively challenging. This article presents a case from the Department of Urology at Guiqian International General Hospital, where a patient with urethral stricture following phalloplasty underwent placement of a thermo-expandable nickel-titanium shape memory alloy stent. The details are reported as follows.

## 2 Clinical data

**Figure 1:**
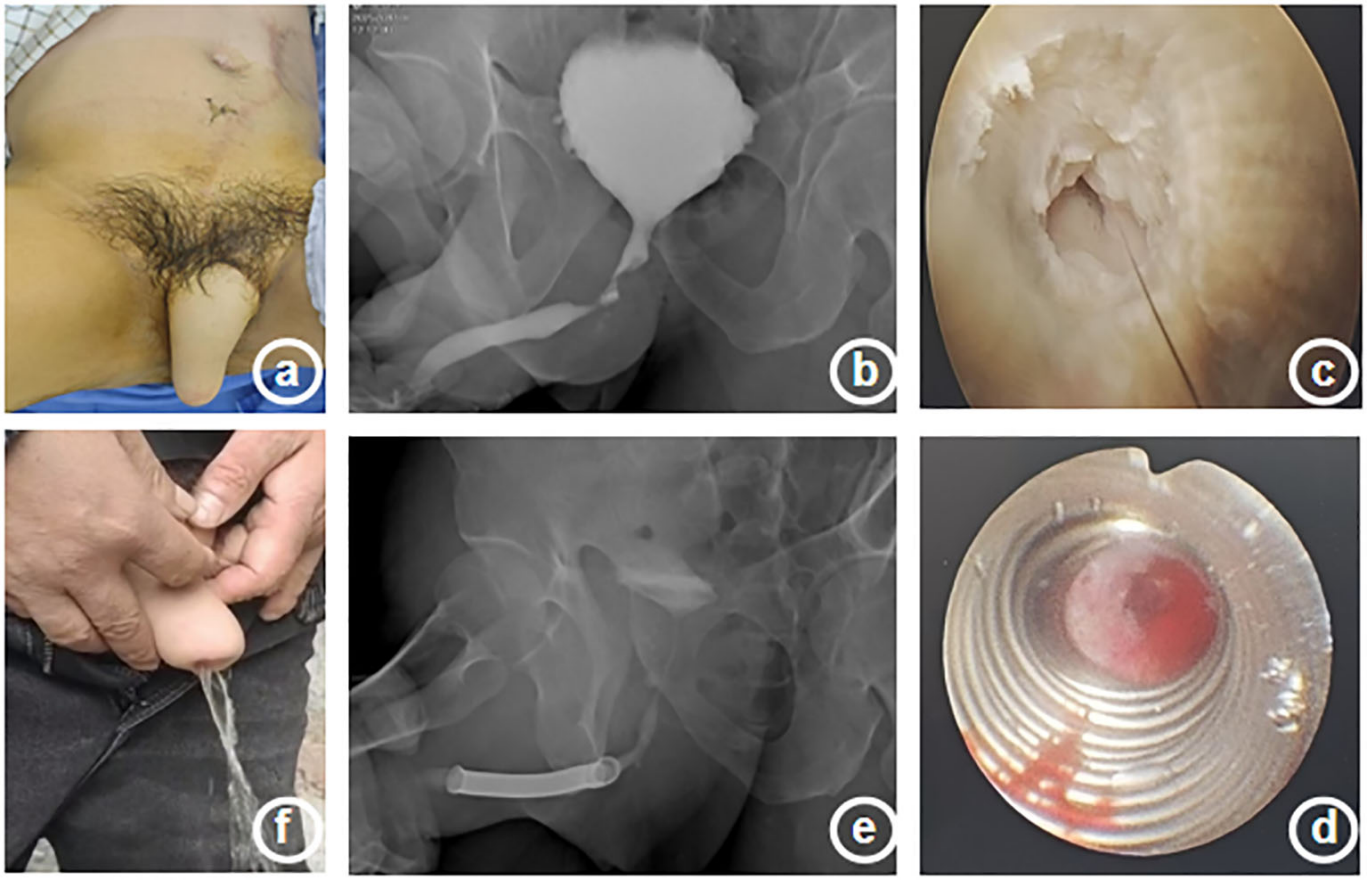
Clinical presentation, imaging findings, intraoperative, and postoperative features of a patient with urethral stricture. **(a)** Postoperative appearance of reconstructed penis **(b)** Schematic diagram of preoperative urethrography **(c)** Urethral stricture during surgery **(d)** Metal stent placed during surgery **(e)** Schematic diagram of postoperative urethrography **(f)** Postoperative urination condition.

**Figure 2:**
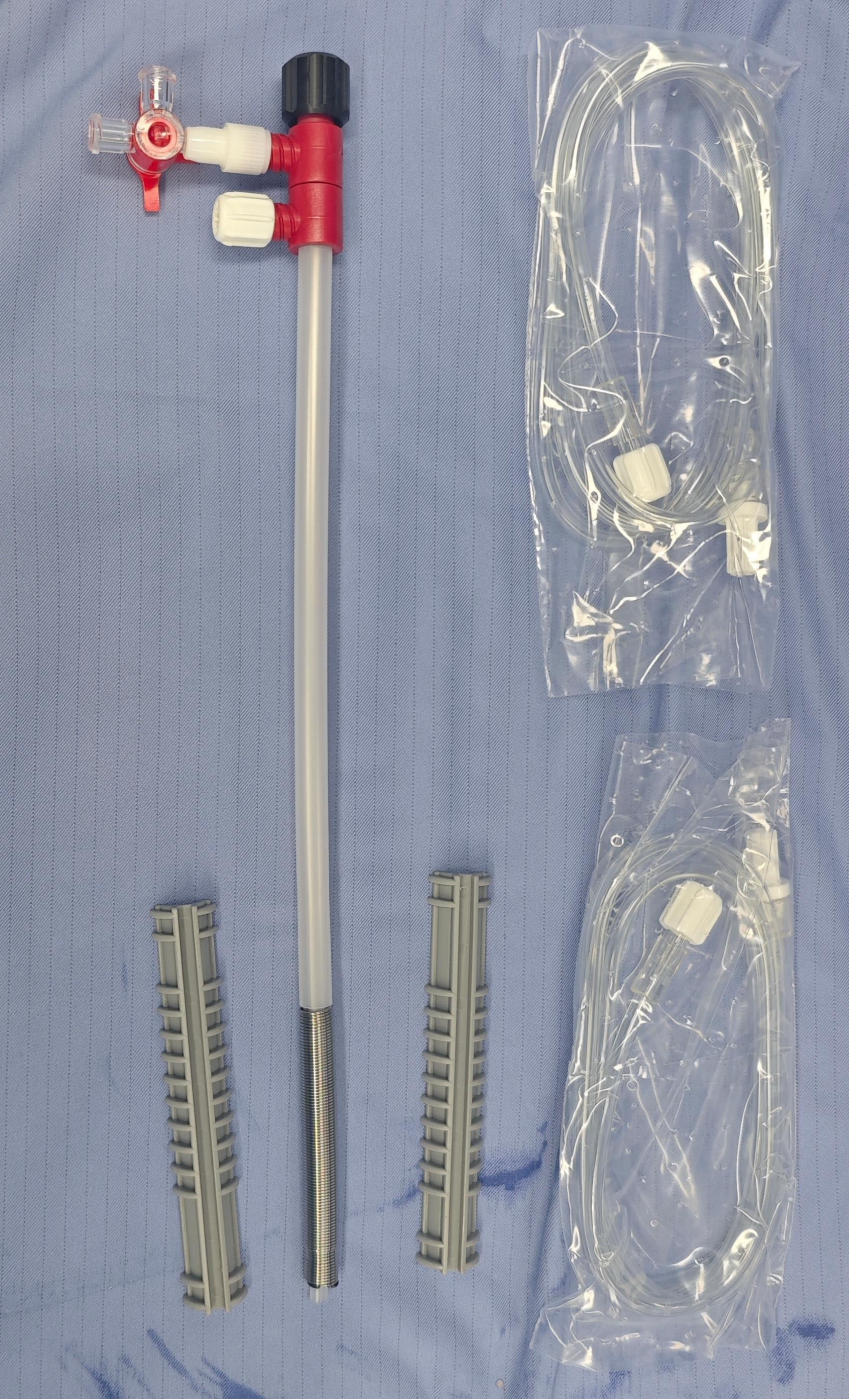
Appearance diagram of urethral metal stent.

The patient, a 51-year-old male, was admitted to the hospital due to “dysuria for 14 years, aggravated with fever for 1 day”. Medical history: 20 years ago, the patient underwent lower abdominal island flap phalloplasty at another hospital due to penile trauma. Dysuria occurred 6 years after the surgery. The patient underwent repeated urethral dilation treatments, but exhibited poor response. Physical examination revealed a visible old surgical scar on the left abdomen and postoperative appearance of the phalloplasty (**Figure 1a**). Urethrography after admission demonstrated localized stricture at the anastomotic site of the short urethra in the reconstructed penis (**Figure 1b**). Urethrocystoscopy showed multiple trabeculae formation in the bladder and stenosis was seen at the urethral anastomosis (**Figure 1c**). The patient requested the placement of a thermo-expandable nickel-titanium shape memory alloy metal stent. Following urethral dilation under general anesthesia, a 26Fr cystoscope was inserted transurethrally. A urethral metal stent kit was deployed, and a 5 cm urethral metal stent (**Figure 2**) was placed under direct visualization, with the proximal end approximately 2 cm from the urethral sphincter. The stent fully covered the anastomotic stricture and scarred segment of the urethra. The kit was withdrawn, and urethroscopy confirmed proper stent placement (**Figure 1d**). An 8Fr single-lumen urethral catheter was indwelled, and the procedure concluded. One day after the surgery, the urethral catheter was removed, and the patient resumed normal urination (**Figure 1f**) without urinary incontinence or other discomforts. One month after the surgery, the patient returned to the hospital for a follow-up examination, and the urethrography revealed that after the placement of the urethral metal stent, the contrast agent passed smoothly through the previous stricture at the urethral anastomotic site (**Figure 1e**). During the follow-up period, the patient had unobstructed urination without adverse complications such as weak urine stream or urinary incontinence.

## 3 Discussions

Phalloplasty is a crucial surgical approach to address urinary and sexual function needs in patients with penile defects caused by trauma, tumor resection, or congenital deformities, while also improving their psychological well-being. The main surgical techniques include the tubed flap method, pedicled flap method, free flap method, Cheng’s method, and composite free flap method (1, 2). Urethral stricture is a common complication following phalloplasty (3, 4). Its pathogenesis is primarily associated with the characteristics of urethral reconstruction materials (mainly skin grafts): compared to native urethral mucosa, the thin epithelial layer and thick dermal layer of skin are more prone to significant scar formation. Poor healing and scar contracture serve as the fundamental causes of urethral stricture.

Urethral stricture may lead to severe complications such as hydronephrosis, recurrent urinary tract infections, and urinary retention. The current treatment options include periodic urethral dilation, urethroplasty, cystostomy, perineal urethrostomy, etc. However, the application of metal urethral stents for the management of urethral strictures secondary to phalloplasty has not been reported either in domestic or foreign literature, and there is currently a lack of unified guidelines or consensus for its diagnosis and treatment.

The Memokath™ third-generation spiral stent is made of nickel-titanium shape memory alloy and features unique thermodynamic properties: It expands and reshapes to anchor within the urethra when exposed to 55°C warm water. Unlike early mesh stents, its spiral structure minimizes tissue overgrowth *in vivo*. When cooled in 5°C ice water, the stent contracts and softens, which facilitates removal. The urethral stent offers advantages such as a relatively simple implantation procedure, high survivorship, excellent wear resistance, superior safety profile, and fewer complications (5). It has been applied in treating voiding dysfunction caused by benign prostatic hyperplasia (6) and prostate cancer (7). The patient in this case experienced long-term recurrent urethral stricture following phalloplasty, and showed poor response to conventional dilation therapy. We utilized the Memokath™ thermo-expandable nickel-titanium shape memory alloy stent for treatment, with preliminary results demonstrating its effectiveness in relieving reconstructed urethral anastomotic strictures and restoring unobstructed urination. During short-term follow-up (up to the submission date), no occurrences of common complications such as stent migration, urinary incontinence, or recurrent infections were observed. Compared to repeated urethral dilation or urethroplasty requiring complex flap repair, this minimally invasive approach may offer a novel and less traumatic treatment option for specific patients, which avoids the pain associated with repeated procedures and the uncertainty surrounding therapeutic outcomes.

According to literature review, this case represents the first reported instance in China of successfully placing a thermo-expandable nickel-titanium shape memory alloy stent (Memokath™) under direct cystoscopic vision for the management of urethral stricture following phalloplasty. This case has provided preliminary confirmation of the feasibility and short-term efficacy of this technology. With advancements in minimally invasive urological techniques and the emergence of novel devices, there have been more treatment options available for complex urethral strictures (including reconstructed urethral strictures). However, its long-term safety, efficacy, and applicable populations still require further evaluation through studies with larger sample sizes and extended follow-up.

## Data Availability

The original contributions presented in the study are included in the article/supplementary material. Further inquiries can be directed to the corresponding author.

## References

[1] Garaffa G Sansalone S Ralph DJ . Penile reconstruction. Asian J Androl. (2013) 15:16–9. doi: 10.1038/aja.2012.9, PMID: 22426595 PMC3739136

[2] Yulin D Wensen X Shuzhong G . Selection of surgical methods for phalloplasty. J Clin Surg. (2016) 24:171–2.

[3] Esmonde N Bluebond-Langner R Berli JU . Phalloplasty flap-related complication. Clin Plast Surg. (2018) 45:415–24. doi: 10.1016/j.cps.2018.03.017, PMID: 29908631

[4] Yede Z Xudong L Gang L Qiang Z Qinglian H . Experience in the management of urethral stricture following phalloplasty. J Navy Med. (2010) 31:242–3.

[5] Sedigh O Dalmasso E Barale M Pizzuto G Agosti S Dadone C . UVENTA urethral stents: Are we taking a step forward? The first clinical series. Arch Esp Urol. (2021) 74:435–40., PMID: 33942736

[6] Cerrato C Antoniou V Somani BK . Prostatic stents: a systematic review and analysis of functionaloutcomes and complication rate. Prostatic Dis. (2025) 28:318–27. doi: 10.1038/s41391-024-00915-y, PMID: 39516581

[7] Choi SY Lim B Chi BH Kim JH Lee W Kyung YS . Efficacy and tolerability of metallic stent in patients with Malignant prostatic obstruction secondary to prostatic cancer. Low Urin Tract Symptoms. (2021) 13:329–34. doi: 10.1111/luts.12367, PMID: 33768708

